# Active IR Thermography Evaluation of Coating Thickness by Determining Apparent Thermal Effusivity

**DOI:** 10.3390/ma13184057

**Published:** 2020-09-12

**Authors:** Alexey Moskovchenko, Vladimir Vavilov, Michal Švantner, Lukáš Muzika, Šárka Houdková

**Affiliations:** 1 New Technologies—Research Centre, University of West Bohemia, Univerzitní 8, 301 00 Plzeň, Czech Republic; alexeym@ntc.zcu.cz (A.M.); muzika@ntc.zcu.cz (L.M.); houdkov@ntc.zcu.cz (Š.H.); 2 School of Nondestructive Testing, Tomsk Polytechnic University, 30, Lenin Avenue, 634050 Tomsk, Russia; vavilov@tpu.ru; 3 Faculty of Mechanics and Mathematics, Tomsk State University, 30, Lenin Avenue, 634050 Tomsk, Russia

**Keywords:** pulse thermography, coating thickness, apparent effusivity, thermal NDT, thermally sprayed coatings, thermographic testing, infrared non-destructive testing

## Abstract

Pulsed thermography is a common technique for nondestructive testing (NDT) of materials. This study presents the apparent effusivity method for the quantitative evaluation of coating thickness in a one-sided thermal NDT procedure. The proposed algorithm is based on determining a threshold value of apparent effusivity, which can be found for particular coating-on-substrate structures. It has been found that the square root of the time at which the apparent effusivity curve reaches this threshold is proportional to the coating thickness. The efficiency of the proposed approach is demonstrated by analytical modeling and experimentation performed on thermally-sprayed coatings.

## 1. Introduction

Coatings are covering layers that are applied to the surface of materials, also called substrates. Various kinds of coatings are widely used for restoration and enhancement of the lifetime of metallic constructions by protecting them from corrosion and wear [1,2]. Coatings are differed by purposes, materials, and application procedures. Coating thickness is an important parameter used for the evaluation of coating quality and service life. Therefore, nondestructive testing (NDT) methods allowing determination of coating thickness are required in the manufacture and operation of materials with coatings. Coating thickness can be evaluated by using numerous NDT techniques, such as eddy-current, X-ray, ultrasound, Terahertz, etc. [3,4]. Each technique is characterized by certain advantages and limitations. The eddy-current and ultrasound methods are precise but require contact with materials to be tested and calibration on many test samples. The X-ray method is also appropriate and illustrative, but it cannot often be used because of safety reasons and possible material damage caused by X-rays. Terahertz testing is time-consuming and requires expensive equipment.

Infrared thermography is a perspective alternative to other commonly used methods since it is non-contact, fast, and illustrative [5]. It is based on the evaluation of surface thermal radiance of test objects, or temperature, by using infrared (IR) cameras in either active or passive test procedures. Active thermography requires external thermal stimulation, typically heating, of the tested object. It can be classified in respect of a heat source (optical, eddy-current, ultrasonic, etc.) and a type of modulation of a heat function in time (flash, “square” pulse, step, and thermal wave heating) [6]. There have been a lot of IR thermographic test procedures and data processing algorithms suggested for evaluating coating thickness [7]. Thermal contrast methods [8] require choosing a reference point (area) that is suitable for defect detection but may be difficult for coating thickness quantification if heating is uneven. Pulse phase and lock-in thermographic techniques allow finding a correlation between the Fourier phase and coating thickness, but the corresponding relationships are complicated, like a fourth-order polynomial [9]. The analysis of the second logarithmic derivatives [10], often called thermographic signal reconstruction (TSR), is sensitive and reveals a clear “thickness-peak derivation time” dependency. However, the presence of noise and necessity to apply polynomial fitting, as well as finite duration of heating, may affect the accurate determination of derivative maximums or even add some “false” peaks in derivative evolution. Typical processing algorithms were comparatively analyzed elsewhere [11,12] to show that some improvements can be achieved by manipulating temperature evolutions in time or applying principal component analysis (PCA) [13]. However, the corresponding algorithms are often time-consuming or having no clear physical meaning; also, the results of applying such algorithms may be affected by a number of noise factors.

In this study, we apply the apparent thermal effusivity technique for the evaluation of coating thickness. The method is based on a particular physical model intended for the determination of material thermal properties and defect characterization. Unlike the approaches described in [14,15,16,17], where the apparent effusivity technique was used for defect detection and thermal property measurement, we apply this method for quantitative evaluation of coating thickness. We suggest using a threshold value of apparent effusivity that is a combination of the effusivities of the coating and substrate. This technique is non-contact, reference-free, and sensitive. The most important limitation of this technique is the fact that the thermal effusivity of the substrates and coatings should be fairly different to have a clear transient between measured effusivities of two layers. However, this is a typical requirement for all thermal NDT methods. The measurable thickness range is limited by the influence of the finite heat pulse from one side and heat losses from the other. In this study, we demonstrate the method for measuring the thickness of Cr 13% Fe thermally-sprayed coatings with thickness in the 0.1–1.1 mm range. The analysis of limitations and performance of the proposed method for other types of coating-surface structures, in particular, thick and low-conductive, requires further research.

## 2. Apparent Thermal Effusivity Implementation

The apparent thermal effusivity technique is based on the classical heat conduction solution describing 1D temperature distribution in a semi-infinite adiabatic body after the Dirac-pulse heating [18]:

(1) $$T\left(z,t\right)=\frac{Q_{0}}{\sqrt{\rho Ck\pi t}}\exp\left(-{\left(\frac{z}{\sqrt{4\alpha t}}\right)}^{2}\right)$$

where *Q*_0_ is the energy absorbed by the surface as a result of flash heating, *α* = *k*/(C*ρ*) is the thermal diffusivity, *ρ* is the density, *C* is the heat capacity, *k* is the thermal conductivity, *z* is the in-depth coordinate. By introducing the thermal effusivity $e=\sqrt{\rho Ck}$, Equation (1) can be written for surface temperature *T*(0,*t*) in the well-known form [19]:

(2) $$T\left(0,t\right)=\frac{Q_{0}}{e\sqrt{\pi t}}$$

The effusivity can be expressed as:

(3) $$e\left(t\right)=\frac{Q_{0}}{T\left(0,t\right)\sqrt{\pi t}}$$

**Figure 1:**
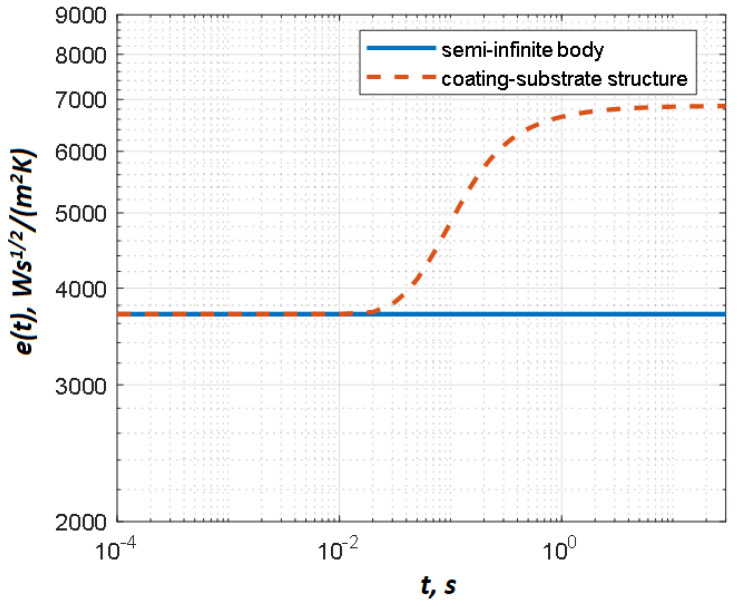
Determining thermal effusivity by Equations (3) and (4).

For a semi-infinite homogeneous body, the curve *e(t)* is a straight line parallel to the abscissa axis with the ordinate axis value equal to $e=\sqrt{\rho Ck}$ (see Figure 1). In real cases, the thermal effusivity calculated by Equation (3) is a function of time being affected by heat pulse duration, heat exchange with the ambient, and finite thickness of the tested sample. For a coating-substrate structure (two-layer material) surface temperature can be expressed [14]:

(4) $$T\left(0,t\right)=\frac{Q_{0}}{e\sqrt{\pi t}}\left(1+2\overset{+\infty }{\underset{n=1}{\sum }}{\left(-R\right)}^{n}\right)\exp\left(-\frac{n^{2}d^{2}}{\alpha t}\right)$$

where *d* is the coating thickness, *e_c_* and *e_s_* are the effusivities of the coating and substrate respectively, $R=\frac{e_{s}-e_{c}}{e_{s}+e_{c}}\;$ is the reflection coefficient at the boundary between the coating and substrate.

In practice, absorbed energy can hardly be determined, therefore, the concept of the pixel-based apparent thermal effusivity was proposed [15,16]:

(5) $$e_{app}\left(L,t\right)=\frac{1}{T\left(L,t\right)\sqrt{t}}$$

Non-stationary heat conduction is the process of diffusion of thermal energy. In the 1D case, the time behavior of the surface temperature is related to the in-depth coordinate *z* by the known expression [20]:

(6) $$z=\sqrt{\pi \alpha t}$$

In other words, deeper material layers appear on the body surface with greater time delays. The same phenomenon can be observed by analyzing the time-evolution *e(t)*. As illustrated in Figure 1, the initial section of the *e(t)* curve represents the thermal effusivity of the coating material, and then, after a certain transition period, it goes to the substrate thermal effusivity. The inflection point in the *e(t)* curve corresponds to the depth of the first layer and the ratio between the thermal properties of the coating and substrate. Hence, coating thickness can be evaluated by analyzing the corresponding inflection points. Note that, if the absorbed energy is unknown, the effusivity *e(t)* can be replaced by the apparent effusivity *e_app_(t)* characterized by the same time behavior.

It is difficult to identify inflection points from real experimental data because of noise, the influence of heat pulse duration, and spurious reflected radiation. It has been demonstrated [15,16] that variations in coating thickness lead to a simple translation of the effusivity curve along the time axis (in the logarithmic representation). Hence, the apparent effusivity curve for any coating-substrate structure (Figure 1) crosses a particular value between the coating and substrate effusivities, while variations in coating thickness shift the time point of this intersection. In this study, the use of the so-called threshold time *t*_t_ is suggested. Such threshold time corresponds to the point where the effusivity curve crosses a particular threshold value located between the values of the coating and substrate effusivities. The *t*_t_ values and their relationship to coating thickness can be found either experimentally or by modeling particular test situations (see below).

## 3. Analytical Model

**Figure 2:**
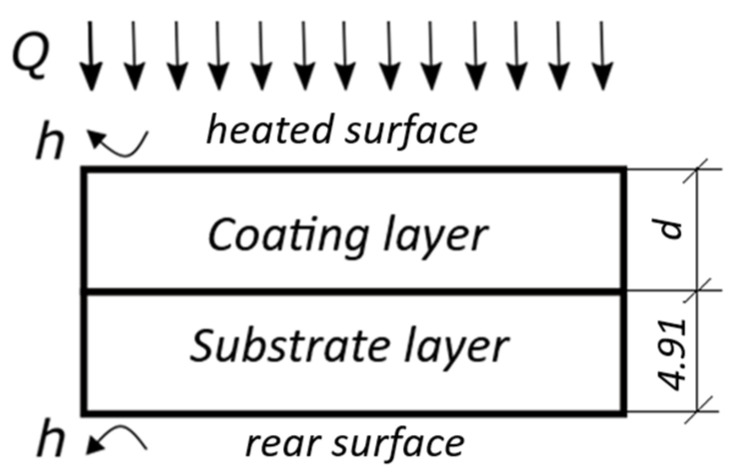
Coating model.

The modeling was performed by using the Thermix-Analytic-3L software from Tomsk Polytechnic University (Version 2.0, Tomsk, Russia). The software implements a 1D semi-analytical solution for heating a three-layer non-adiabatic plate with a square pulse by using the method of thermal quadrupoles described in [21]. In our case, the model represented a two-layer sample heated by a 6 ms-long square pulse with the heat power density of 1.6 × 10^6^ W/m^2^ (Figure 2). The inter-layer boundary conditions were negligible including continuity of the boundary temperatures and heat fluxes, and the body’s initial temperature was zero. The heat exchange coefficients *h* on both the front and rear surfaces were 10 W m^−2^ K^−1^ to specify the non-adiabatic heat conduction problem. The thermal properties of the coating and substrate are presented in Table 1 (averaged by the published data). Seven values of coating thickness corresponded to the used experimental samples. The calculated result represented temperature evolutions on the front surface with the time step of 0.001 s (up to 3000 images in total).

## 4. Experimental Setup

Two experimental 70 × 25 mm samples included a 4.91 mm-thick steel substrate with the Cr 13% Fe coatings produced by twin-wire arc spraying (TWAS) technology [23]. This technology is based on arc melting of two cored wires, which are fed into the system. The Cr 13% Fe coatings produced by the TWAS technology are characterized by high heterogeneity, high amounts of porosity, oxide inclusions, and inter-splat boundaries [24]. The expected thermal conductivity of such coating is from 2 to 10 W·m^−1^·K^−1^ [24]. The substrate was made from a S235 construction steel, of which thermal conductivity was assumed to be in the range from 40 to 50 W·m^−1^·K^−1^. It was expected that the combination of materials with the above-mentioned thermal properties and coating thickness from 0.1 to 1 mm should provide detectable temperature signals when applying pulsed IR thermography [25].

**Figure 3:**
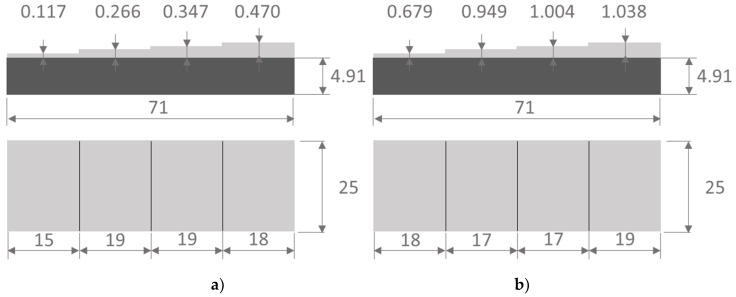
Scheme of sample 1 of coating thickness in the range 0.117–0.470 mm (**a**) and sample 2 of coating thickness in the range 0.679–1.038 mm (**b**).

The scheme of the samples is shown in Figure 3. The first sample contained four coating areas with thicknesses of 0.117 (d1), 0.266 (d2), 0.347 (d3), and 0.470 (d4) mm. Respectively, the second sample contained four coating areas with thicknesses of 0.679 (d5), 0.949 (d6), 1.004 (d7), and 1.038 (d8) mm. The lateral dimensions of the coated areas were 18 × 25 mm.

**Figure 4:**
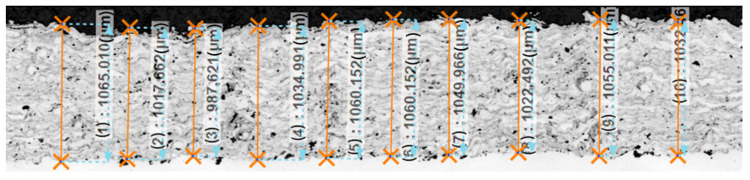
Validating coating thickness by microscopic measurements (mean value 1.038 mm).

The surface of the coatings remained in the as-sprayed state, without any further treatment. Therefore, the samples were fairly rough thus providing some uncertainty and ambiguity in the measurement of coating thickness. In some selected areas (cut cross-sections), the coating thickness was validated by ten microscopic measurements, and the mean value was assumed as characterizing coating thickness, see Figure 4.

The heating of the samples was performed with a single Hensel EH Pro 6000 flash tube, (including flash lamp HD 9450143, 9-inch diameter reflector, and cooling fan, made by HENSEL) providing a 6 ms/6 kJ optical pulse and 6 ms pulse, and IR thermograms were captured by a FLIR A6751 IR imager (made by FLIR Systems, Inc., Wilsonville, OR, USA) at the frame rate of 400 Hz (2000 frames images in a sequence). The measurement setup and the principle of synchronization between the flash-lamp and camera were described in detail elsewhere [25]. Since the samples were small enough, they were tested at once, and the effect of uneven heating was considered negligible.

Background subtraction and median filter with a 3 × 3 spatial mask were applied to all IR thermograms in the captured sequence before further processing.

## 5. Results and Discussion

### 5.1. Modeling Results

**Figure 5:**
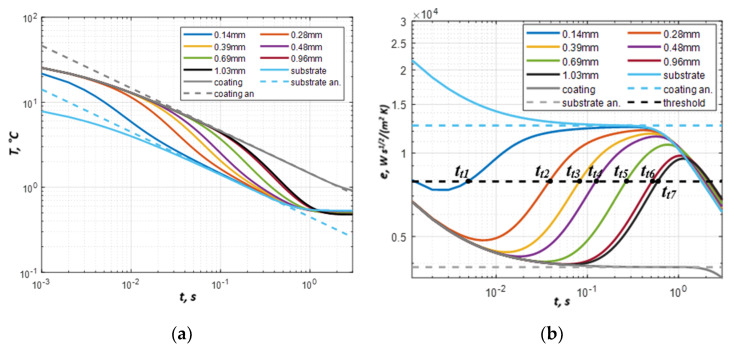
Determining thermal effusivity by modeling: temperature (**a**) and apparent effusivity (**b**) evolution.

Computed temperature and diffusivity evolutions are shown in Figure 5a,b. The dashed lines represent the temperature evolution of the semi-infinite bodies made of coating and substrate materials respectively after having applied a Dirac pulse (Equation (2)). Solid lines represent the results obtained by analytical modeling for coatings of different thicknesses. There is a certain discrepancy between semi-infinite (Equation (2)) and simulated temperature evolution at the beginning and at the end of the thermal process. This is caused by the finite duration of the heat pulse and heat exchange with the ambient. The effusivity curves in Figure 5b were obtained by applying Equation (3) to the temperature data in Figure 5a. For the pure coating and the pure substrate, the effusivity evolutions *e(t)* tend to the true effusivities with the above-mentioned deviations at the beginning and the end of the process. Thus, only a limited section of the corresponding curves, where the plateau is observed, should be used for determining thermal effusivity. This section is illustrated in Figure 5b (0.1–0.4 s interval), where the effusivity curves of the coating and substrate match the real effusivity value (dashed lines).

**Figure 6:**
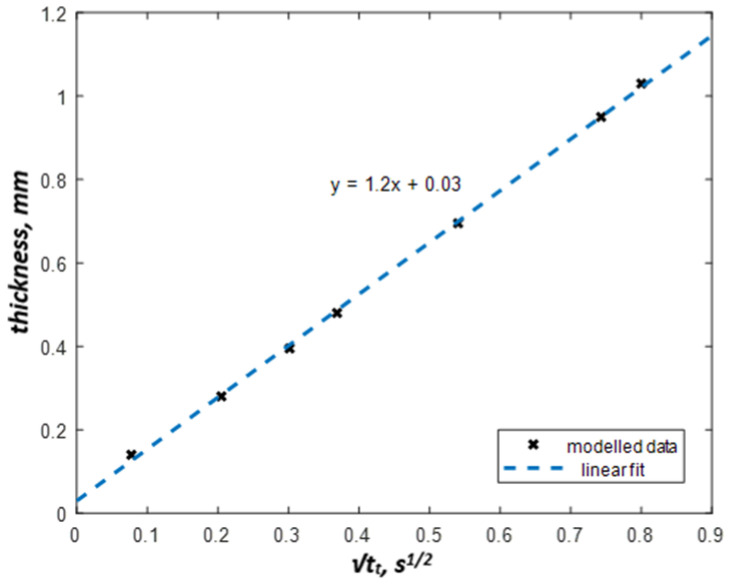
Square root of threshold time vs. coating thickness (modeling).

The effusivity evolution of the coating-substrate structure first follows the curve of the coating effusivity, and afterwards, it tends to the substrate effusivity. However, the effusivity curves do not reach the substrate’s true effusivity value obtained for the semi-infinite body. An effusivity threshold can be set from a range of values between coating and substrate effusivities. However, it should intersect all effusivity curves in the considered range of coating thicknesses. Figure 6 shows that there is a linear relationship between the coating thickness and the square root of the threshold time. Thus, any threshold within the above-mentioned range can be used. In our analysis of modeling results, we have used, for example, the value:

*e_t_* = (*e_c_* + *e_s_*)/2 (7)

It leads to the effusivity threshold value of *e_t_* = 0.79 × 10^4^ W∙s^1/2^ m^−2^ K^−1^. The respective threshold times can be then obtained by an intersection of the effusivity threshold line and apparent effusivity curves of the coatings with different thicknesses (see *t_t1_* – *t_t7_* in Figure 5b).

The result of the analytical modeling illustrates the basic concept of coating thickness determination based on the evaluation of the effusivity threshold and threshold time. The corresponding linear dependence can be used for the determination of both coating thickness and its variations. A correct effusivity threshold can be found analytically by known thermal properties of materials. However, in real experiments, material thermal properties, as well as input energy, are mostly unknown. Therefore, the effusivity threshold has to be estimated experimentally using reference materials and an appropriate calibration procedure. The experimental effusivity evolution *e(t)* (Equation (3)) can be replaced with its replica evolution *e_app_(t)* (Equation (5)) thus requiring no knowledge of absorbed energy.

### 5.2. Experimental Results

**Figure 7:**
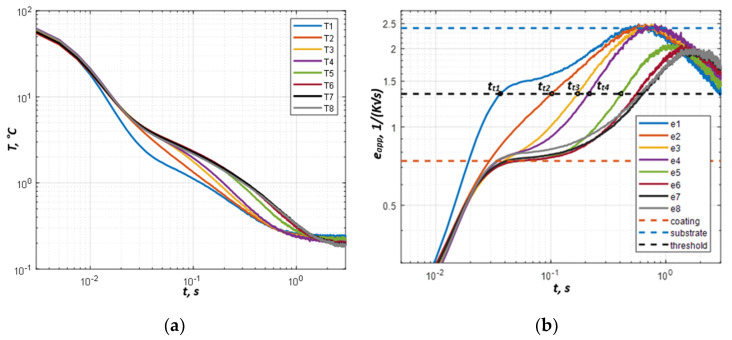
Experimental evolution of temperature (**a**) and apparent effusivity (**b**) for steel sample with Fe 13% Cr TWAS coating (Figure 2).

The temperature evolutions T1–T8 (Figure 7a) were averaged with a 50 × 50 mask in each coating area d1–d8 and processed by applying Equation (5) for the calculation of apparent effusivity evolutions presented in Figure 7b. The curves obtained show that, unlike modeling results in Figure 5, the experimentally determined apparent effusivity values increase from lower to higher values at the beginning of the process, where the corresponding analytical curves dropped down. This means that the temperature decreases faster than predicted by the modeling at the beginning (up to about 0.03 s), probably, because of the contribution of reflected radiation to the measured temperature signal, which is supposed to sharply decrease after the flash. However, experimental and modeling results get closer after approximately 0.03 s.

The effusivities of the coating and substrate were determined as the plateau values in Figure 7b (dashed lines). Because of noise, the determining of an effusivity threshold between two plateau values is difficult. We have obtained “optimal” threshold values by trial and error in the following form:

(8) $$e_{t}=\mathrm{Exp}\left(\frac{\ln e_{app\;c}+\ln e_{app\;s}}{2}\right)$$

**Figure 8:**
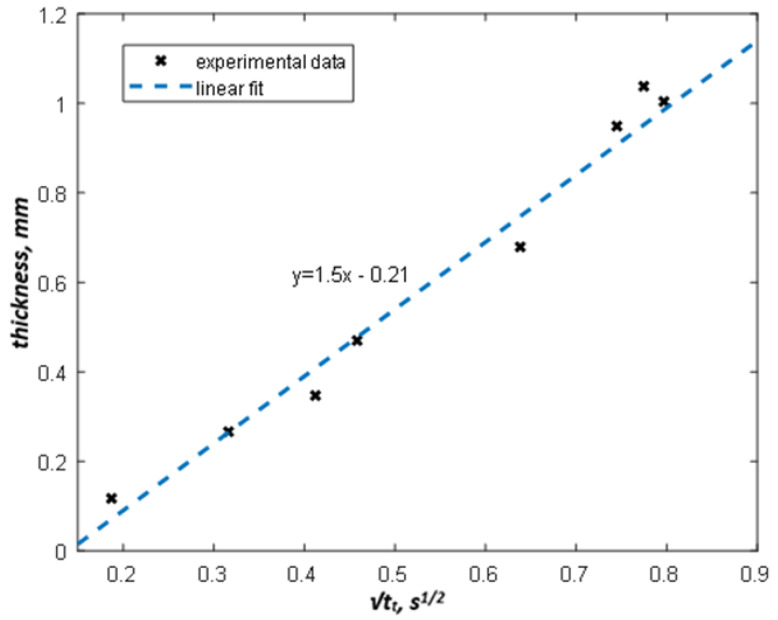
Experimental dependence of square root of threshold time vs. coating thickness.

Figure 8 shows the $\sqrt{t_{t}}$ vs. coating thickness dependence with the 1.34 (K^−1^ s^−1/2^) threshold value obtained by Equation (8).

Based on the linear fitting of the data in Figure 8, the equation for coating thickness dependence on the threshold time has been obtained in the form:

(9) $$d=1.5\sqrt{t_{t}}-0.21$$

**Figure 9:**
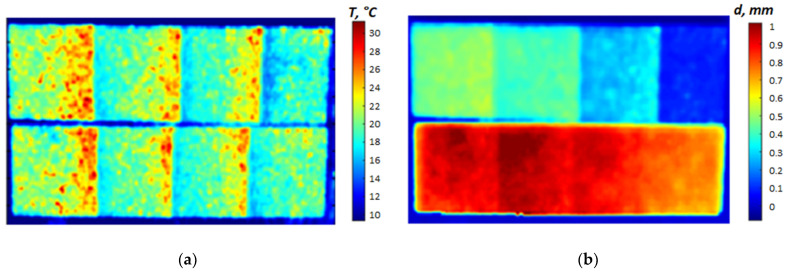
Raw IR thermogram at 10 ms after the flash (**a**) and map of coating thickness (**b**).

**Figure 10:**
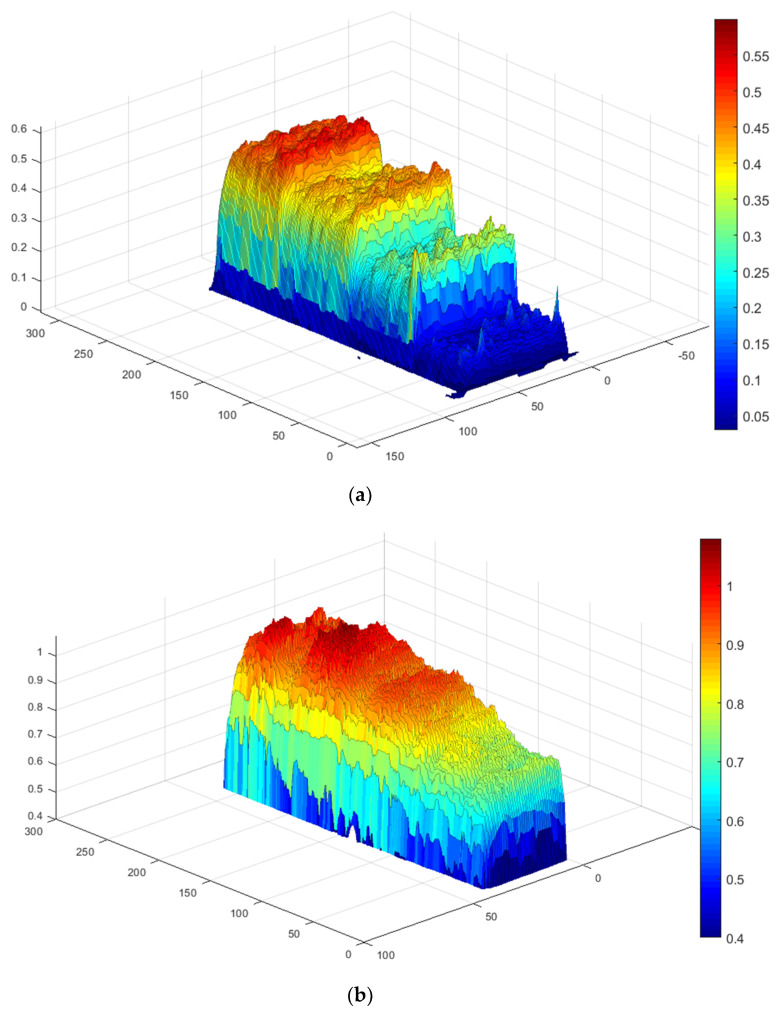
Three-dimensional coating thickness maps: (**a**) sample 1, (**b**) sample 2.

The standard deviation of the regression above is σ = 36.68 µm. Equation (9) represents a linear calibration function with the coefficients depending on the thermal properties of coatings and substrates, as well as the chosen effusivity threshold. The above-mentioned procedure allows determining the calibration relationship for any reference sample and producing the corresponding coating thickness map by using an appropriate conversion of raw temperature data. This approach is illustrated by the example in Figure 9. More examples of coating thickness maps in samples 1 (*d* = 117, 266, 347 and 470 µm) and 2 (*d* = 679, 949, 1004, 1038 µm) are presented in Figure 10 in a 3D presentation.

The proposed approach can also be useful in the evaluation of coating thickness variations. Table 2 contains the mean values of coating thickness in d1–d8 areas, as well as the corresponding absolute and relative differences and standard deviations, obtained by both micrometric and thermographic measurements. The micrometric data were averaged by 10 point measurements while IR thermographic results were obtained within the 50 × 50 pixel areas. The maximum relative error (20.5%) corresponded to the minimum thickness, and the maximum absolute difference (119 µm) appeared in the thickest coating. Since the calculation procedure is based on a linear fitting of micrometric measurements, the discrepancy in the results follows the deviations between the experimental data and the linear regression (Figure 8). Note that micrometry was performed at single points while IR thermography provided results across the whole surface being affected by non-uniform properties of the coating (oxide inclusions, porosity, uneven absorption, etc.).

## 6. Conclusions

The study proposed a flash thermography technique intended for the determination of coating thickness based on using the apparent thermal effusivity concept and threshold observation time. Unlike other approaches, for example, those using an inflection point in the apparent effusivity evolution curve, the threshold observation time was defined as a crossing point of the apparent thermal effusivity curve and a pre-defined effusivity threshold. A linear dependence between the square root of the threshold time and coating thickness was predicted by the theoretical analysis, thus allowing to produce 2D distributions of absolute coating thickness, as well as its variations. Compared with other coating characterization approaches, the suggested technique is simple and robust in application to practical test cases.

The theoretical concept was verified by numerical modeling and experiments conducted on Cr 13% Fe coatings. The coatings were deposited on steel substrates using the TWAS thermal spraying technology with the coating thickness varying from 0.1 to 1 mm. The analytical modeling and the experiments confirmed a linear dependence between the thickness of the coatings and the square root of the threshold time with the latter one being determined by the logarithmic coordinates as a mean value of the substrate and coating effusivities.

The IR thermographic results were validated by the micrometric measurement of the coating thickness resulting in the average difference between measured values from 13 to 119 µm and the standard deviation of about 40 µm. Such characterization accuracy is considered acceptable for practical purposes, since thermal effusivity measurements are affected by a number of factors, such as unknown absorbed energy, finite heat pulse duration, heat exchange with the ambient, coating roughness, uneven absorption, etc. Addressing these factors and studying the limitations of the proposed method might be the subject for further research.

## Figures and Tables

**Table 1:** Material thermal properties used in modeling.

Parameter	Coating	Substrate *
Thickness *d*, mm	0.117, 0.266, 0.347, 0.470, 0.679, 0.949, 1.038	4.91
Conductivity, W∙m∙K^−1^	6.12	40
Diffusivity, m^2^ s^−1^	2.74 × 10^−6^	1.11 × 10^−5^
Effusivity, W∙s^1/2^ m^−2^ K^−1^	0.37 × 10^4^	1.21 × 10^4^

* Steel S235 [22].

**Table 2:** Coating thickness measurement results.

Area	Micrometry Thickness Measurement, µm			Thermography Thickness Measurement, µm			Difference, µm/%
	Min/Max Value	Mean	Standard Deviation	Min/Max Value	Mean	Standard Deviation	
d1	77/176	117	37.7	71/179	93	12.5	−24/−20.5
d2	222/334	266	40.6	227/371	283	24.9	17/6.4
d3	281/408	347	37.5	361/486	414	19.2	67/19.3
d4	422/507	470	24.9	409/551	483	19.6	13/2.8
d5	617/735	679	39.1	661/818	747	26.3	68/10
d6	914/972	949	17.6	751/969	876	45.6	−73/−7.7
d7	948/1058	1004	39.4	818/1076	954	44.5	−50/−5
d8	987/1065	1038	24.6	799/1050	919	42.1	−0.119/−11.5
